# Effect of boundary slips and magnetohydrodynamics on peristaltic mechanism of Jeffrey nanofluid along with microorganisms through a porous medium

**DOI:** 10.1016/j.heliyon.2024.e33949

**Published:** 2024-07-02

**Authors:** Arshad Riaz, Muhammad Dil Nawaz, Muhammad Naeem Aslam, Sami Ullah Khan, Shafiq ur Rehman, Ghaliah Alhamzi

**Affiliations:** a Department of Mathematics, Division of Science and Technology, University of Education, Lahore, 54770, Pakistan; b School of Mathematics, Minhaj University, Lahore, Pakistan; c Department of Mathematics, Namal University, Mianwali, Pakistan; d Department of Mathematics, University of Chakwal, Chakwal, Pakistan; e Department of Mathematics and Statistics, College of Science, Imam Mohammad Ibn Saud Islamic University (IMSIU), Riyadh, Saudi Arabia

**Keywords:** Porous medium, Jeffery-nanofluid, Entropy generation, Vicious dissipation, Peristaltic flow, Slip boundary, Thermal slip

## Abstract

The development on entropy generation in fluid flows has applications in many medical equipment such as cryogenic devices and therapeutic heat apparatus. This study looks at how porous medium, multi-slips, and entropy formation affect the pumping of Jeffrey nanofluid flow through an asymmetric channel containing motile microorganims. A lubrication theory is used to neglect the fluctuation effects in the flow. Numerical simulations are utilized to generate data for specific physical features of the problem utilizing the Shooting approach on Mathematica. Following a thorough research, it is appropriate to conclude that the porous medium's permeability reduces flow speed along the walls while increases at the center of the flow region. Graphical representation of the results further reveals that entropy production can be decreased by including high thermal slip and low viscous slip elements. It is also worth noting that the Brinkman number reduces the thermal distribution in the flow while it helps in increasing the flow speed.

## 1 Introduction

In literature review, the term nanofluid elaborated in many ways, but many of them concluded that it is mixture of nanoparticles in which the particles of 100 nm has scattered in the liquid. To increase the amount and stability of nanofluid and the thermal diffusivity Dey et al. [1] presented a comprehensive analysis for various thermo-physical properties. Heyhat et al. [2] described the impact of viscosity and density on a nanolayer by technique of computational modeling of molecular dynamics and found that the basic liquid decreased due to nanolayer formation. Menni et al. [3] conducted a number of effective investigations about nanofluids in three fundamental complex geometries: heat exchangers, solar energy collectors, and microchannels. They had contributed to a number of ways by providing the different experimental and numerical methods. Some significant metrics were used for numerical and experimental result like heat flux, pressure drop and coefficient of heat transfer with single and double phase included. Each factor was checked on the basis of proposed mechanisms which could increase or decrease the performance. Nanoparticles enhance heat transmission, better oil recovery, lubrication, and medication due to their higher heat transfer, wettability and rheological characteristics [4]. In accordance with theoretical predictions, Zhang et al. [5]'s research indicates that initial consistency decreased with increasing nanofluid concentrations. This feature held true for the first eight days of testing, providing valuable new insights into the theory of nanofluid stability and advancing the field of nanofluid technology. Hamzat et al. [6] investigated the role of nanofluids in a variety of solar-powered dialysis structures, solar ovens, vacuum tube solar panels, solar dishes, parabolic funnel solar collectors, rectangular plate solar collectors, directly absorbing solar collectors, and photoelectric thermal systems. Anjum et al. [7] was to examine the gyrotactic microbes, a bioconvection mechanism, and the dependable three-dimensional flow of Cross nanofluid. The behavior of motile micro bacteria was demonstrated graphically and established outcomes. Mebarek-Oudina et al. [8] used numerical analysis to evaluate the magnetite–water nanofluid hydromagnetic flow that arises from a rotating stretchable disk. The nanofluid flow was represented by the modified Buongiorno model, which took into consideration the primary slip mechanisms and the effective nanofluid attributes that depend on the volume fraction. Some more recent works on nanofluids can be cited in Refs. [[9], [10], [11], [12], [13]].

Non-Newtonian Jeffrey fluids exhibit unique rheological properties. Viscosity varies with stress direction and pace, as demonstrated by mixtures of water and cornstarch that solidify rapidly after being agitated vigorously. Theoretical models based on Jeffery fluid properties explain such phenomena in fluid dynamics and polymer production. The three-dimensional Jeffrey fluid flow passing through a porous stretched sheet was examined by Hayat et al. [14]. They found that the velocity trend decreased as the ratio of relaxation to retardation times increased. Greater thermal relaxation values were also associated with a decrease in temperature distribution. Soomro et al. [15] established heat transmission along a stretching surface and non-Newtonian nanofluid flow. It was observed that the zero flux caused the nanoparticles concentration to scatter at the boundary layer surface. Contributions from MHD, porous layer and elasticity of the fluid resulted in a thinner boundary layer, while the magnetic field and porous enhanced the temperature distribution of the flow domain. Saleem et al. [16] examined MHD Jeffrey fluid movement through a perpendicular cone with heat effect. They examined the chemical and thermal sinks. Furthermore, consistent concentration and heat flux conditions were applied. It was found that the heat sink and source had variations in the temperature boundary layer breadth. Khan et al. [17] determined the MHD flow mixture of fluids, with respect to a time-dependent expanding sheet. Using a moving endoscope, Haroun et al. [18] analyzed the impact of a varying electric strength on Jeffrey transport along a heated, vibrating cylindrical tube. They observed that when an electric field existed, the impact of fraction of breadth and Jeffry factor on velocity axis were more pronounced and evident than when the electric field was removed. A continual spontaneous laminar inertial Jeffrey fluid motion over a stretching surface has been investigated by Nisar et al. [19]. Moreover, the chemical procedure and a constant thermal source increased transportation of temperature and mass of the non-Newtonian fluid. Importantly, they noted that enhancing the interaction factor heat flux of fluid falls down for rising Hartmann factor but for smaller values reverse impact is shown and they also estimated that the velocity is improving by both interaction factor and Hartmann number. The effects of temperatures, asymmetric thermal rays, and Brownian motions on MHD motion for Jeffrey fluid were examined by Dharmaiah et al. [20]. It concern with the flow of simulation reactor. Dakin-Gari et al. [21] investigated heat, mass analysis for flow of Jeffrey fluid model under magnetic effect through a porous heated material. They found that the mass transfer rate increased along with the Peclet number. It was found that the mass diffusion rate decreased with the chemical reaction.

Peristalsis is the rhythmic contraction and expansion of a tube that moves fluid in one direction, much like food passes down the esophagus. It is crucial in several domains such that Peristaltic pumps are used by many industries to precisely measure fluids for tasks like mining and chemical processing, guaranteeing a smooth and effective flow of liquids without compromising the purity of the final product. Precise sample handling and contamination-free drug delivery are beneficial to medicine. Akram et al. [22] worked to investigate the peristaltic study considering double diffusion scenario for magnetic field impact on Prandtl nanofluid along with thermal radiations. The key finding include that the nanoparticles volume fraction falls against the Prandtl number. Sinnott et al. [23] replicated duodenal peristalsis taking a suspension of stiff particles in a viscous fluid. They found a non-homogeneous solids distribution along the duodenum as a result of these pushing events. By adding solids, the overall propulsive flow and retrograde jet were marginally altered. When there were no restrictions from solids or tissue, the viscoelastic tube wobbles transversely. The effect of variable viscosity on the peristaltic flow of the Jeffrey fluid model, which was taken to be a nanofluid with an asymmetric channel, was examined by Hasona et al. [24]. This concept was initial cause of development in the peristaltic field. The results for oil molecules showed that increasing temperatures provided them more energy, which satisfied the primary objective of refining crude oil to provide them with more velocity. Mansour et al. [25] investigated the temperature and energy transfer effects on the wavy flow of a Williamson fluid model in the space among concentric cylinders. The data gathered indicated that temperature increased in tandem with increases in the Eckert and Weissenberg numbers. However, a distinct pattern was observed when comparing the concentration to the temperature. Rashid et al. [26] discussed an incompressible Williamson fluid in a curved conduit having magnetic effect with peristaltic motion. The contribution of variable factors in evaluating flow of blood in constrictive veins was examined by Rajashekhar et al. [27]. In particular, they investigated the change in viscosity and heat conductivity, which contributed to clarifying the rheological properties of biological fluids such as blood, urine, and eye drops. They found that the velocity Newtonian fluid was higher than non-Newtonian fluid. The impact of dual diffusive convection on peristalsis flow of magneto nanofluid through a symmetric non-uniform conduit was examined by Alhazmi et al. [28]. The knowledge gained from current work was very impressive for creating magneto peristalsis pumping which used for drug delivery phenomena and particularly for heat. The peristalsis mechanism for Jeffrey liquid under a conduit with porous medium was studied by Rafiq et al. [29] produced the characteristics of Jeffrey's fluid similar to the blood. Elmhedy et al. [30] mainly addressed the impact of thermal exchange and magnetic fields on the pumping flow of the Rabinowitsch fluid stresses in an inclined channel. They examined the magnetic field impact on heat exchange during peristalsis. Some recent studies on peristaltic procedure can be found in Refs. [[29], [30], [31], [32], [33]].

Entropy generation in energy processes measures the irreversibility caused by friction and heat transmission. Car engines serve as examples of how energy is used to evaluate a system's efficiency when part of it is converted to work and the rest is wasted heat, increasing entropy. The efficiency of turning heat into power is improved by thermoelectric devices, particularly when waste heat is involved. Rashidi et al. [34] looked at the entropy of third-order nanofluid slip flow through a flexible plate embedded in a porous plate. Applications of heat transfer, convective boundary conditions, nanoparticle density, and non-Fourier heat flux in zero mass flux were investigated. Yusuf et al. [35] studied the entropy generation rate for MHD Williamson nanofluid flow through an inclined convectively heated extensible plate. Throughout their investigation, porous material, heat radiation, and chemical reactions were all taken into consideration. It was very first time when Li et al. [36] described Entropy analysis for improvement of super capacitor. The permanent changes in the supper capacitor cell taken due to heat transfer, ohmic loss and mass exchange were correctly quantified by the entropy generation. In this way they determined the efficiency of mechanisms that was impossible to find by traditional energy analysis. Zhao et al. [37] investigated the material of the combustor wall, the inlet velocity, and the inlet equivalency ratio—three critical variables that affect a micro-combustors thermal performance. It was illustrated that silicon carbide had better thermal performance when the combustor wall material was changed by increasing the stander wall temperature and continuity of combustor wall temperature. Moreover, it did not appear that compositional changes to the combustor wall would have a significant impact on the heat loss resulting from entropy formation. Researchers studying cavity flows would do well to consult the work of Rosca et al. [38], which sought to statistically investigate the constant natural convective heat transfer of hybrid particles within an imperfectly heated trapezoidal shape region with linear temperature distributions at tilted walls, caused by the consistent Lorentz force. This will help researchers better understand the flow topologies and nature of hybrid nanofluid properties. To find potential regimes with low entropy formation rates, a thorough generation of entropy investigation was also carried out.

A porous media is a material with interconnected void spaces, or pores, through which liquids or gases can move. These materials can be either natural (soil, sand, or rock) or manmade (porous ceramics or polymers). Krishna et al. [39] looked into the impacts of Hall and ionic drift on the MHD convective movement of elastico-viscous fluid across a media with pores between two opposing plates with a time-dependent sinusoidal pressure gradient and a rigid rotation. It was demonstrated that speed fell with increasing elastico-viscous factor and Hartman number. The momentum boundary layer's thickness was increased by the Schmidt number, while the velocity was decreased by the thermal radiation parameter. The characteristics of tangent hyperbolic fluid movement along an extensible sheet within a porous medium were examined by Alkaoud et al. [40]. The examination's key conclusions were that temperature rises and fluid velocity decreases with increases in the power-law index, slippage parameter, porous factor, and magnetic number. In the study of Zhang et al. [41], it was noted that while there were considerable spatial and temporal differences in the temperature rise, the MRI signal variations diminished. The expected results of the study might have had a significant influence on the optimal way to optimize gas hydrate phase transition. When Reddy et al. [42] addressed the MHD features that influence the heat exchange of an incompressible viscous material numerically across a constantly expanding horizontal cylinder submerged in a porous media with an internal heat sink or production; they demonstrated the importance of these properties. It was anticipated that the temperature gradient in the boundary layer region surrounding the cylinder would worsen with a rise in its porosity factor and curvature parameter. To enhance the energy conversion and efficiency of hydrogen-powered combustion, a miniature recuperative planar with porous media was proposed. As a result, the addition of porous media raised the temperature of the burner radiation, and the size and porosity of the porous media affected the burner's thermal performance, lowering the temperature of the exhaust gas and increasing energy efficiency [43].

Gyrotactic microorganisms, like bacteria or algae, are driven by factors like gravity or fluid flow to assist them navigate and move in cyclic or spiral patterns according to environmental stimuli. Gyrotactic plankton in aquatic environments moves vertically or in circles to optimize exposure to sunlight and minerals while avoiding predators and allows them to adjust its location in response to variations in light and nutrients. Hosseinzadeh et al. [44] explored cross-fluid motion having gyrotactic microbes and nanoparticles on a level, 3-D cylinder with magnetic effect and viscous force. They discovered that there was an about 78.38 % decrease in the quantity of microorganisms when the Lewis number increased from 0.25 to 0.55. A dimensionless simulation of bio convective motion of gyrotactic bacteria was described by Avramenko et al. [45]. The study conducted by Xia et al. [46] examined the 3D nonlinear hybrid convective flow in the boundary layer of micropolar nanofluid hybrids under different slip conditions and in the case of microorganisms over the thinning surface. The main topic of Wang et al.'s study [47] was the fluid of random 3-D Maxwell biological convective microscopic units moving in the direction of an exponentially expanding surface when influenced by an organic response slip condition. They found that the slip parameter for velocity, as it rose in both directions, was responsible for the decreasing velocity component behavior. Hui et al. [48] used the Direct Simulation Monte Carlo (DSMC) method to study the velocity slips and heating fluctuation for a two-dimensional rough plate under hypersonic conditions. Compared to the traditional first-order slip boundary conditions, they found that modifying the slip effect improved the accuracy of tiny particles parameter, especially the factor of temperature conduction.

After a thorough examination of the existing literature, the authors found that the combination of peristaltic flow, motile microbes, porous medium, and magnetic field offers a promising pathway for improving nutrient transport, biofilm development, and pollutant cleanup in a variety of environmental and biomedical applications. It is observed that a very few researches have been existed about viscoelastic fluids with gyrotactic microbes performing peristaltic motion under heat and velocity slip circumstances both inside a porous medium and with a magnetic field. This paper investigates computational solutions for peristaltic transport of Jeffrey nanofluid inside a vertical conduit containing gyrotactic microbes under speeds and temperature slip boundary conditions. It includes an examination of entropy owing to viscosity temperature impacts using a bio-convective effect. There is also worth noting that examines temperature transport using thermal line as opposed to only producing 2-D graphs. The investigation is scientifically confined by applying a mechanical engineering the theory of lubrication that assumes a high length of wave and a low Reynolds number. The numerical answers are computed using the shooting method, which incorporates computational software Mathematica and the NDSolve tool. A full graphical description is provided for the parametric influence of relevant factors on different quantities of the problem.

## 2 Geometric description and mathematical model

Here, we investigate an attributes for a Jeffery nanofluid containing gyrotactic microorganisms in an asymmetric porous channel along with magnetohydrodynamic (MHD). A sinusoidal wave propagates over the channel walls at a steady velocity ${\;c}_{1}$. The symbol λ represents the wavelength of peristaltic waves. Constant values of heat are $T_{1}$, $T_{0}$ are at the leftward and rightward surfaces of the channel [49].

An expression of two wavy surfaces in mathematics for an asymmetric channel are given as

(1) $$\begin{array}{c} {\bar{H}}_{1}=d_{1}+a_{1}\;\cos\left[\frac{2\pi }{\lambda }\left(\bar{X}-c_{1}\bar{t}\right)\right]\text{,}\\ {\bar{H}}_{2}=-\left(d_{2}+b_{1}\;\cos\left[\frac{2\pi }{\lambda }\left(\bar{X}-c_{1}\bar{t}\right)+\omega \right]\right)\text{.} \end{array}$$

Here, the symbols $a_{1}$ and $b_{1}$ indicate the wave amplitudes, the parameter ω shows a deviation in the phase angle. In elements of the flow field that are considered as $\left(\bar{U},\bar{V}\right)$, here are the statically-framed governing expressions [49,50]

(2) $$\frac{\partial \bar{U}}{\partial \bar{X}}+\frac{\partial \bar{V}}{\partial \bar{Y}}=0\text{,}$$

(3) $$\frac{\partial \bar{U}}{\partial \bar{t}}+\bar{U}\frac{\partial \bar{U}}{\partial \bar{X}}+\bar{V}\frac{\partial \bar{U}}{\partial \bar{Y}}=-\frac{1}{\rho_{f}}\frac{\partial \bar{P}}{\partial \bar{X}}+\frac{1}{\rho_{f}}\frac{\partial {\bar{S}}_{X\;X}}{\partial \bar{X}}+\frac{1}{\rho_{f}}\frac{\partial {\bar{S}}_{XY}}{\partial \bar{Y}}-\frac{\sigma B_{0}^{2}}{\rho_{f}}\bar{U}-\left(\frac{1}{1+\lambda_{1}}\right)\frac{\mu }{\bar{K}}\bar{U}+\frac{g}{\rho_{f}}\left(\begin{array}{c} \left(1-\varphi_{1}\right)\rho_{f}\beta \left(\bar{T}-{\bar{T}}_{0}\right)-\\ \left(\rho_{p}-\rho_{f}\right)g\left(\bar{C}-{\bar{C}}_{0}\right)-\left(\bar{n}-{\bar{n}}_{0}\right)\gamma_{1}\Delta \rho \end{array}\right)\text{,}$$

(4) $$\frac{\partial \bar{V}}{\partial \bar{t}}+\bar{U}\frac{\partial \bar{V}}{\partial \bar{X}}+\bar{V}\frac{\partial \bar{V}}{\partial \bar{Y}}=-\frac{1}{\rho_{f}}\frac{\partial \bar{P}}{\partial \bar{Y}}+\frac{1}{\rho_{f}}\frac{\partial {\bar{S}}_{YX}}{\partial \bar{X}}+\frac{1}{\rho_{f}}\frac{\partial {\bar{S}}_{YY}\;}{\partial \bar{Y}}-\left(\frac{1}{1+\lambda_{1}}\right)\frac{\mu }{\bar{K}}\bar{V}\text{,}$$

(5) $$\begin{array}{c} \frac{\partial \bar{T}}{\partial \bar{t}}+\bar{U}\frac{\partial \bar{T}}{\partial \bar{X}}+\bar{V}\frac{\partial \bar{T}}{\partial \bar{Y}}=\alpha \left(\frac{\partial^{2}\bar{T}}{\partial {\bar{X}}^{2}}+\frac{\partial^{2}\bar{T}}{\partial {\bar{Y}}^{2}}\right)+\\ \tau \left(\begin{array}{c} D_{B}\left(\frac{\partial \bar{C}}{\partial \bar{X}}\frac{\partial \bar{T}}{\partial \bar{X}}+\frac{\partial \bar{C}}{\partial \bar{Y}}\frac{\partial \bar{T}}{\partial \bar{Y}}\right)\\ +\frac{D_{T}}{{\bar{T}}_{0}}\left({\left(\frac{\partial \bar{T}}{\partial \bar{X}}\right)}^{2}+{\left(\frac{\partial \bar{T}}{\partial \bar{Y}}\right)}^{2}\right) \end{array}\right)+\left(\begin{array}{c} {\bar{S}}_{XX}\frac{\partial \bar{U}}{\partial \bar{X}}+{\bar{S}}_{XY}\frac{\partial \bar{V}}{\partial \bar{X}}+\\ {\bar{S}}_{YX}\frac{\partial \bar{U}}{\partial \bar{Y}}+{\bar{S}}_{YY}\frac{\partial \bar{V}}{\partial \bar{Y}} \end{array}\right)-\left(\frac{1}{1+\lambda_{1}}\right)\frac{\mu }{\bar{K}}\left({\bar{U}}^{2}+{\bar{V}}^{2}\right), \end{array}$$

(6) $$\frac{\partial \bar{C}}{\partial \bar{t}}+\bar{U}\frac{\partial \bar{C}}{\partial \bar{X}}+\bar{V}\frac{\partial \bar{C}}{\partial \bar{Y}}=D_{B}\left(\frac{\partial^{2}\bar{C}}{\partial {\bar{X}}^{2}}+\frac{\partial^{2}\bar{C}}{\partial {\bar{Y}}^{2}}\right)+\frac{D_{T}}{{\bar{T}}_{0}}\left(\frac{\partial^{2}\bar{T}}{\partial {\bar{X}}^{2}}+\frac{\partial^{2}\bar{T}}{\partial {\bar{Y}}^{2}}\right)\text{,}$$

(7) $$\frac{\partial \bar{n}}{\partial \bar{t}}+\bar{U}\frac{\partial \bar{n}}{\partial \bar{X}}+\bar{V}\frac{\partial \bar{n}}{\partial \bar{Y}}=\frac{bW_{c}}{\left({\bar{C}}_{1}-{\bar{C}}_{0}\right)}\left(\frac{\partial }{\partial \bar{Y}}\left(\bar{n}\frac{\partial \bar{C}}{\partial \bar{Y}}\right)+\frac{\partial }{\partial \bar{X}}\left(\bar{n}\frac{\partial \bar{C}}{\partial \bar{X}}\right)\right)+D_{m}\left(\frac{\partial^{2}\bar{n}}{\partial {\bar{X}}^{2}}+\frac{\partial^{2}\bar{n}}{\partial {\bar{Y}}^{2}}\right)\text{,}$$

Here, the aforementioned equations are converted into a wave frame using the appropriate transformations stated as

(8) $$\left\langle \bar{u},\bar{v},\bar{x},\bar{y},p\left(\bar{x},\bar{y}\right)\right\rangle =\left\langle \bar{U}-c_{1},\bar{V},\bar{X}+c_{1}\bar{t},\bar{Y\;},P\left(\bar{X},\bar{t}\right)\right\rangle \text{.}$$

To obtain a more precise description of the preceding equations, it is a very important step in fluid mechanics problems to obtain a non-dimensional system of equations. In this scenario, the following conversion rules are used to produce dimensionless quantities.

(9) $$\bar{x}=\frac{\lambda x}{2\pi },\bar{y}=d_{1}y,\bar{u}=c_{1}u,\bar{v}=c_{1}\delta v,\bar{p}=\frac{\mu c_{1}\lambda }{2\pi d_{1}^{2}}p,{\bar{s}}_{xx}=\frac{\mu c_{1}}{d_{1}}s_{xx},\delta =\frac{2\pi d_{1}}{\lambda },d=\frac{d_{1}}{d_{2}},Re=\frac{\rho_{f}c_{1}d_{1}}{\mu },\bar{T}={\bar{T}}_{0}+\left({\bar{T}}_{1}-{\bar{T}}_{0}\right)\theta ,\bar{C}={\bar{C}}_{0}-\left({\bar{C}}_{1}-{\bar{C}}_{0}\right)\varphi ,Nr=\frac{\left(\rho_{p}-\rho_{f}\right)\left(C_{1}-C_{0}\right)g\varphi }{\left(1-\varphi_{1}\right)\left(T_{1}-T_{0}\right)\rho_{f}\beta },\bar{n}={\bar{n}}_{0}-({\bar{n}}_{1}-{\bar{n}}_{0)}\gamma ,Pe=\frac{bW_{c}}{D_{m}},Rb=\frac{\gamma \Delta \bar{n}\Delta \rho }{\rho_{f}\beta \Delta T\;},{\;M}^{2}=\frac{d_{1}^{2}B_{0}^{2}\sigma }{\mu },\mathrm{Pr}=\frac{\nu }{\alpha },G=\frac{\left(1-\varphi_{1}\right)\left({\bar{T}}_{1}-{\bar{T}}_{0}\right)g\beta d_{1}^{2}}{\mu c_{1}},Br=\frac{{\mu c}_{1}^{2}}{\alpha \left({\bar{T}}_{1}-{\bar{T}}_{0}\right)},K=\frac{\bar{K}}{d_{1}^{2}}\text{.}$$

Using above given transformations in Eqs. (2), (3), (4), (5), (6), (7), we get

(10) $$\frac{\partial u}{\partial x}+\frac{\partial v}{\partial y}=0\text{,}$$

(11) $$Re\delta \left(u\frac{\partial u}{\partial x}+v\frac{\partial u}{\partial y}\right)=-\frac{\partial p}{\partial x}+\delta \frac{\partial s_{xx}}{\partial x}+\frac{\partial s_{xy}}{\partial y}-\left(M^{2}+\left(\frac{1}{1+\lambda_{1}}\right)\frac{1}{K}\right)\left(u+1\right)+G\left(\theta -Nr\varphi -Rb\gamma \right)\text{,}$$

(12) $$Re\delta^{3}\left(\delta \;u\frac{\partial v}{\partial x}+v\frac{\partial v}{\partial y}\right)=-\frac{\partial p}{\partial y}+\delta^{2}\frac{\partial s_{yx}}{\partial x}+\delta \frac{\partial s_{yy}}{\partial y}-\delta \left(\frac{1}{1+\lambda_{1}}\right)\frac{1}{K}v\text{,}$$

(13) $$\begin{array}{c} PrRe\delta \left(u\frac{\partial \theta }{\partial x}+v\frac{\partial \theta }{\partial y}\right)=\left(\delta^{2}\frac{\partial^{2}\theta }{\partial x^{2}}+\frac{\partial^{2}\theta }{\partial y^{2}}\right)+PrNb\left(\delta^{2}\frac{\partial \varphi }{\partial x}\frac{\partial \theta }{\partial x}+\frac{\partial \varphi }{\partial y}\frac{\partial \theta }{\partial y}\right)+\\ PrNt\left(\delta^{2}{\left(\frac{\partial \theta }{\partial x}\right)}^{2}+{\left(\frac{\partial \theta }{\partial y}\right)}^{2}\right)+Br\left(\delta s_{xx}\frac{\partial u}{\partial x}+\delta s_{xy}\frac{\partial v}{\partial x}+s_{yx}\frac{\partial u}{\partial y}+s_{yy}\frac{\partial v}{\partial y}\right)-\left(\frac{1}{1+\lambda_{1}}\right)\frac{Br}{K}\left({\left(u+1\right)}^{2}+\delta^{2}v^{2}\right)\text{,} \end{array}$$

(14) $$ReSc\left(\delta u\frac{\partial \varphi }{\partial x}+v\frac{\partial \varphi }{\partial y}\right)=\delta^{2}\frac{\partial^{2}\varphi }{\partial x^{2}}+\frac{\partial^{2}\varphi }{\partial y^{2}}+\frac{Nt}{Nb}\left(\delta^{2}\frac{\partial^{2}\theta }{\partial x^{2}}+\frac{\partial^{2}\theta }{\partial y^{2}}\right)\text{,}$$

(15) $$ReSc\delta \left(u\frac{\partial \gamma }{\partial x}+v\frac{\partial \gamma }{\partial y}\right)=\left(\delta^{2}\frac{\partial^{2}\gamma }{\partial x^{2}}+\frac{\partial^{2}\gamma }{\partial y^{2}}\right)-Pe\left(\sigma \left(\delta^{2}\frac{\partial^{2}\varphi }{\partial x^{2}}+\frac{\partial^{2}\varphi }{\partial y^{2}}\right)+\gamma \left(\delta^{2}\frac{\partial^{2}\varphi }{\partial x^{2}}+\frac{\partial^{2}\varphi }{\partial y^{2}}\right)+\left(\delta^{2}\frac{\partial \varphi }{\partial x}\frac{\partial \gamma }{\partial x}+\frac{\partial \varphi }{\partial y}\frac{\partial \gamma }{\partial y}\right)\right)\text{,}$$

Cauchy tensor of stress for Jeffrey fluid [[16], [17], [18], [19], [20], [21]] is described as

(16) $$S=\frac{\mu }{1+\lambda_{1}}\left(A_{1}+\lambda_{2}\frac{dA_{1}}{dt}\right)$$

The following shear stresses are components of Cauchy stress tensor

(17) $${\bar{S}}_{yx}={\bar{S}}_{xy}=\frac{\mu }{1+\lambda_{1}}\left(\frac{\partial \bar{u}}{\partial \bar{y}}+\frac{\partial \bar{v}}{\partial \bar{x}}\right)$$

Assuming a lubrication theory, the non-zero stresses are found as

(18) $$s_{xy}=\frac{1}{1+\lambda_{1}}\left(\frac{\partial u}{\partial y}\right)\text{.}$$

Using Eq. (18) and considering assumptions of long wave length and low Reynolds number, Eqs. (10), (11), (12), (13), (14), (15) become

(19) $$-\frac{\partial p}{\partial x}+\frac{1}{1+\lambda_{1}}\left(\frac{\partial^{2}u}{\partial y^{2}}\right)-\left(M^{2}+\left(\frac{1}{1+\lambda_{1}}\right)\frac{1}{K}\right)\left(u+1\right)+G\left(\theta -Nr\varphi -Rb\gamma \right)=0\text{,}$$

$$\left(\frac{\partial^{2}\theta }{\partial y^{2}}\right)+PrNb\left(\frac{\partial \varphi }{\partial y}\frac{\partial \theta }{\partial y}\right)+PrNt{\left(\frac{\partial \theta }{\partial y}\right)}^{2}+Br\frac{1}{1+\lambda_{1}}{\left(\frac{\partial u}{\partial y}\right)}^{2}$$

(20) $$-\left(\frac{1}{1+\lambda_{1}}\right)\frac{Br}{K}{\left(u+1\right)}^{2}=0\text{,}$$

(21) $$\frac{\partial^{2}\varphi }{\partial y^{2}}+\frac{Nt}{Nb}\left(\frac{\partial^{2}\theta }{\partial y^{2}}\right)=0\text{,}$$

(22) $$\frac{\partial^{2}\gamma }{\partial y^{2}}-Pe\left(\sigma \frac{\partial^{2}\varphi }{\partial y^{2}}+\gamma \frac{\partial^{2}\varphi }{\partial y^{2}}+\frac{\partial \varphi }{\partial y}\frac{\partial \gamma }{\partial y}\right)=0\text{,}$$

In terms of stream functions, Eqs. (19), (20), reduce to

(23) $$\frac{1}{1+\lambda_{1}}\left(\frac{\partial^{4}\Psi }{\partial y^{4}}\right)-\left(M^{2}+\left(\frac{1}{1+\lambda_{1}}\right)\frac{1}{K}\right)\left(\frac{\partial^{2}\Psi }{\partial y^{2}}\right)+G\left(\frac{\partial \theta }{\partial y}-Nr\frac{\partial \varphi }{\partial y}-Rb\frac{\partial \gamma }{\partial y}\right)=0\text{,}$$

(24) $$\left(\frac{\partial^{2}\theta }{\partial y^{2}}\right)+PrNb\left(\frac{\partial \varphi }{\partial y}\frac{\partial \theta }{\partial y}\right)+PrNt{\left(\frac{\partial \theta }{\partial y}\right)}^{2}+Br\frac{1}{1+\lambda_{1}}{\left(\frac{\partial^{2}\Psi }{\partial y^{2}}\right)}^{2}-\left(\frac{1}{1+\lambda_{1}}\right)\frac{Br}{K}{\left(\frac{\partial \Psi }{\partial y}+1\right)}^{2}=0\text{,}$$

The boundary conditions we have dimensionless form with velocity and temperature slips as [51]:

(25) $$\begin{array}{c} \Psi =\frac{F}{2},\frac{\partial \Psi }{\partial y}=-\frac{\beta_{1}}{1+\lambda_{1}}\left(\frac{\partial^{2}\Psi }{\partial y^{2}}\right)-1,\text{at}\;y=h_{1}\text{,}\\ \Psi =-\frac{F}{2},\frac{\partial \Psi }{\partial y}=\frac{\beta_{1}}{1+\lambda_{1}}\left(\frac{\partial^{2}\Psi }{\partial y^{2}}\right)-1,\text{at}\;y=h_{2}\text{.}\\ \;\theta \left(y\right)=\beta_{2}\frac{d\theta }{dy},\varphi =0,\gamma =0\;\text{at}\;y=h_{1}\text{,}\\ \;\theta \left(y\right)=1-\beta_{2}\frac{d\theta }{dy},\varphi =1,\gamma =1\;\text{at}\;y=h_{2}\text{.} \end{array})$$

## 3 Entropy generation

Irreversibility due to certain physical situations of the problem (in wave frame) is described as [[35], [36], [37], [38],^52^].

(26) $$\begin{array}{c} S_{gen}=\frac{k_{f}}{T_{0}^{2}}\left({\left(\frac{\partial \bar{T}}{\partial \bar{x}}\right)}^{2}+{\left(\frac{\partial \bar{T}}{\partial \bar{y}}\right)}^{2}\right)+\frac{1}{T_{0}}\left({\bar{s}}_{xx}\frac{\partial \bar{u}}{\partial \bar{x}}+{\bar{s}}_{xy}\frac{\partial \bar{v}}{\partial \bar{x}}+{\bar{s}}_{yx}\frac{\partial \bar{u}}{\partial \bar{y}}+{\bar{s}}_{yy}\frac{\partial \bar{v}}{\partial \bar{y}}\right)+\\ \frac{1}{T_{0}}\left[\left(\frac{1}{1+\lambda_{1}}\right)\frac{\mu }{\bar{K}}+\sigma B_{0}^{2}{\left(\bar{u}+1\right)}^{2}\right]+\frac{D_{B}}{T_{0}}\left(\frac{\partial \bar{C}}{\partial \bar{x}}\frac{\partial \bar{T}}{\partial \bar{x}}+\frac{\partial \bar{C}}{\partial \bar{y}}\frac{\partial \bar{T}}{\partial \bar{y}}\right)+\frac{D_{B}}{C_{0}}\left({\left(\frac{\partial \bar{C}}{\partial \bar{x}}\right)}^{2}+{\left(\frac{\partial \bar{C}}{\partial \bar{y}}\right)}^{2}\right)\text{,} \end{array}$$

Putting dimensionless parameters on Eq. (26), we obtain

(27) $$\begin{array}{c} \frac{S_{gen}}{S_{G}}=\delta^{2}{\left(\frac{\partial \theta }{\partial x}\right)}^{2}+{\left(\frac{\partial \theta }{\partial Y}\right)}^{2}+\\ \frac{Br}{\Omega }\left(\delta s_{xx}\frac{\partial u}{\partial x}+\delta s_{xy}\frac{\partial v}{\partial x}+s_{yx}\frac{\partial u}{\partial y}+\delta s_{yy}\frac{\partial v}{\partial y}\right)+\\ \frac{Br}{\Omega }\left(\left(\frac{1}{1+\lambda_{1}}\right)\frac{1}{K}+M\right){\left(\;u+1\right)}^{2}+\Gamma \left(\delta^{2}\frac{\partial \varphi }{\partial x}\frac{\partial \gamma }{\partial x}+\frac{\partial \varphi }{\partial y}\frac{\partial \gamma }{\partial y}\right)+\\ \frac{\Gamma \Lambda }{\Omega }\left(\delta^{2}{\left(\frac{\partial \varphi }{\partial x}\right)}^{2}+{\left(\frac{\partial \varphi }{\partial y}\right)}^{2}\right)\text{.} \end{array}$$

The above mentioned non-dimensional factors are given as:

$$S_{G}=\frac{k_{f}{\left({\bar{T}}_{1}-{\bar{T}}_{0}\right)}^{2}}{{{\bar{T}}_{0}}^{2}d_{1}^{2}},\Omega =\frac{\left({\bar{T}}_{1}-{\bar{T}}_{0}\right)}{{\bar{T}}_{0}}\text{,}$$

(28) $$\Lambda =\frac{{\bar{C}}_{1}-{\bar{C}}_{0}}{{\bar{C}}_{0}},\Gamma =\frac{D_{B}{\bar{T}}_{0}\left({\bar{C}}_{1}-{\bar{C}}_{0}\right)}{\left({\bar{T}}_{1}-{\bar{T}}_{0}\right)},N_{s}=\frac{S_{gen}}{S_{G}}\text{.}$$

Applying lubrication hypothesis of greater wavelength and lesser Reynolds number on Eq. (27)

$$N_{S}={\left(\frac{\partial \theta }{\partial Y}\right)}^{2}+\frac{Br}{\Omega \left(1+\lambda_{1}\right)}{\left(\frac{\partial u}{\partial y}\right)}^{2}+\frac{Br}{\Omega }\left(\left(\frac{1}{1+\lambda_{1}}\right)\frac{1}{K}+M\right){\left(\;u+1\right)}^{2}$$

(29) $$+\Gamma \left(\frac{\partial \varphi }{\partial y}\frac{\partial \gamma }{\partial y}\right)+\frac{\Gamma \Lambda }{\Omega }{\left(\frac{\partial \varphi }{\partial y}\right)}^{2}\text{,}$$

After applying stream functions, we get

$$N_{S}={\left(\frac{\partial \theta }{\partial Y}\right)}^{2}+\frac{Br}{\Omega \left(1+\lambda_{1}\right)}{\left(\frac{\partial u}{\partial y}\right)}^{2}+\frac{Br}{\Omega }\left(\left(\frac{1}{1+\lambda_{1}}\right)\frac{1}{K}+M\right){\left(\frac{\partial \Psi }{\partial y}+1\right)}^{2}$$

(30) $$+\Gamma \left(\frac{\partial \varphi }{\partial y}\frac{\partial \gamma }{\partial y}\right)+\frac{\Gamma \Lambda }{\Omega }{\left(\frac{\partial \varphi }{\partial y}\right)}^{2}\text{,}$$

bejan number function can be written as

(31) $$Be=\frac{{\left(\frac{\partial \theta }{\partial Y}\right)}^{2}{\left(\frac{\partial \theta }{\partial Y}\right)}^{2}+\frac{Br}{\Omega \left(1+\lambda_{1}\right)}{\left(\frac{\partial u}{\partial y}\right)}^{2}+\frac{Br}{\Omega }\left(\left(\frac{1}{1+\lambda_{1}}\right)\frac{1}{K}+M\right){\left(\frac{\partial \Psi }{\partial y}+1\right)}^{2}}{+\Gamma \left(\frac{\partial \varphi }{\partial y}\frac{\partial \gamma }{\partial y}\right)+\frac{\Gamma \Lambda }{\Omega }{\left(\frac{\partial \varphi }{\partial y}\right)}^{2}}\text{,}$$

## 4 Numerical results and discussion

**Fig. 1:**
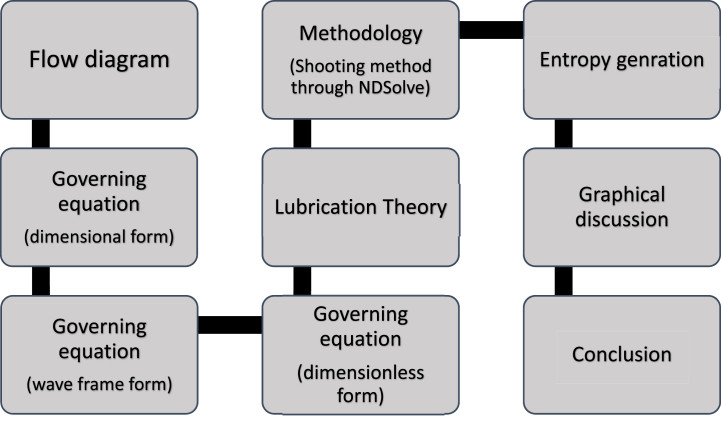
Problem layout.

In Eqs. 21–24, there is a system of complex differential equations with mixed boundary conditions that are non-homogeneous derived from Eq. (25). These kinds of structures are challenging to solve using exact techniques. To deal with situations like this, we depend on either analytical or computational methods. Some methods of analysis are extremely complex, increasing calculation time while simultaneously failing to achieve a suitable convergence rate. Numerical technique, particularly the shooting technique in conjunction with the Runge Kutta 4th order method are more dependable and faster at producing the most exact outcomes. Observing the considerable shortcomings of systematic methods, we chose to employ a numerical algorithm in the current work via the arithmetical tool NDSolve on the computer program Mathematica 11. The entire study's flow chart is shown in Fig. 1 below.

**Fig. 2:**
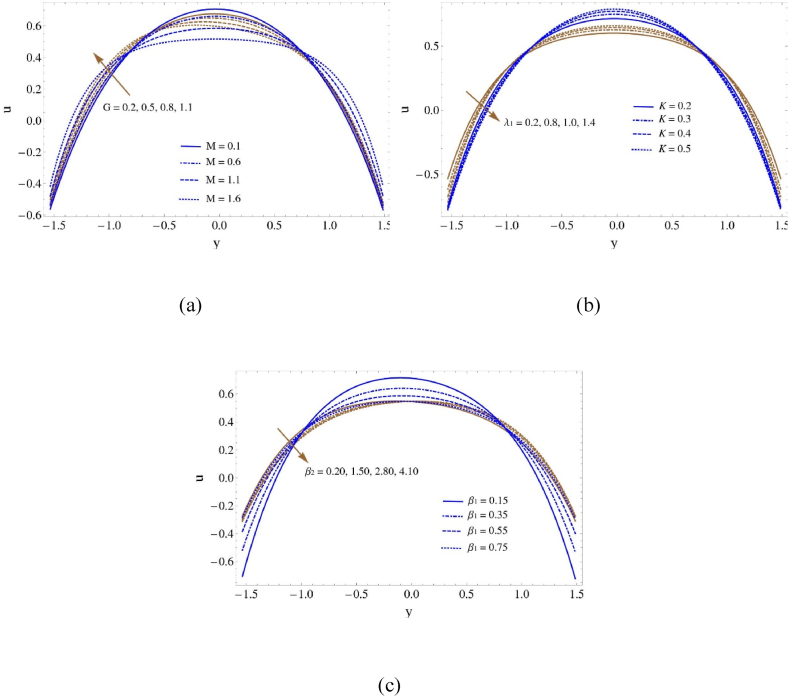
Velosity profile (a) for G and M, (b) for K and $\lambda_{1}$ and (c) for $\beta_{1}$ and $\beta_{2}$

This section further focuses on generating graphs of several relevant characteristics of the current work, including speed, heat, nanoparticle density, microbe concentration, entropy analysis, Bejan factor, temperature contour, and velocity contour. Fig. 2 displays the impact of various variables on velocity: the Hartmann number (M), Grashof number (G), retardation times $\left(\lambda_{1}\right)$ Darcy number (K), boundary slip characteristic $\left(\beta_{1}\right)$, and thermal slip characteristic $\left(\beta_{2}\right)$. The velocity curves in Fig. 2(a) climb on the left and right sides while drop in the center as Hartmann number (M), and Grashof number (G) grow. The ratio of electromagnetic attraction to viscous forces in a conducting fluid flow in the presence of a magnetic field is described by the Hartmann number (M). In comparison to viscous forces, electromagnetic forces become increasingly significant as M grows. This results in a reduction of velocity close to the walls in a magnetohydrodynamic (MHD) flow because of the Lorentz force, which opposes fluid motion perpendicular to the magnetic field lines. The observed climbing of velocity curves is the result of the velocity increasing on the sides where the Lorentz force is less significant. The buoyant forces to viscous forces ratio in a fluid flow due to temperature gradient-induced density fluctuations is quantified by the Grashof number (G). Compared to viscous forces, buoyancy effects become increasingly important as G grows. Buoyancy causes fluid motion in natural convection flows, which are typified by large G values. This motion causes fluid plumes to rise near warm surfaces and sink in cooler locations. This results in a velocity profile where the velocity decreases in the center due to fluid buildup and decreased flow, and increases on the sides due to convective motion away from the heated or cooled surfaces. According to Fig. 2(b)–as retardation times $\left(\lambda_{1}\right)$ increases, the velocity profile raises in the section −1<y<1 and falls in the region where y<−1 and y>1. As the retardation times increase, the velocity profile rises in the section −1<y<1 and lowers in the region where y<−1 and y>1. Retardation times are typically used to characterize the unique timescales associated with the relaxation of the fluid flow due to various physical processes, such as diffusion or viscosity. A rise in $\left(\lambda_{1}\right)$ signifies a reduced fluid flow response to variations in velocity. In the region where the velocity profile rises, −1<y<1, the fluid may adjust to changes in flow conditions more quickly. The velocity rises as a result of this. Conversely, in the regions where y<−1 and y>1, the fluid's delayed response results in a decrease in velocity. With regard to the second parameter Darcy number (K) a drop results in an increase in height for −0.7<y<0.7 and a decrease in the velocity profile for y<−0.7 and y>0.7. A drop in the Darcy number (K) results in an increase in height and a decrease in the velocity profile for y<−0. 7 and y>0.7. The dimensionless Darcy number in a porous medium flow represents the ratio of viscous forces to inertial forces. A decrease in K indicates that viscous forces have a relatively larger effect than inertial forces. As a result, the velocity profile increases near the boundary (−0.7<y<0.7), where the flow is more affected by viscous factors. However, because viscous forces predominate, the velocity profile decreases as one move away from the limit (y<−0.7 and y>0.7). Fig. 2(c) demonstrates that, while $\left(\beta_{2}\right)$ behaves in various ways, velocity lines decrease with a boost in $\left(\beta_{1}\right)$ throughout the range y<−1 and y>1, and increase between them. It follows that with a high value of $\left(\beta_{2}\right)\text{,}$ velocity decreases until it reaches zero and then increases once more.

**Fig. 3:**
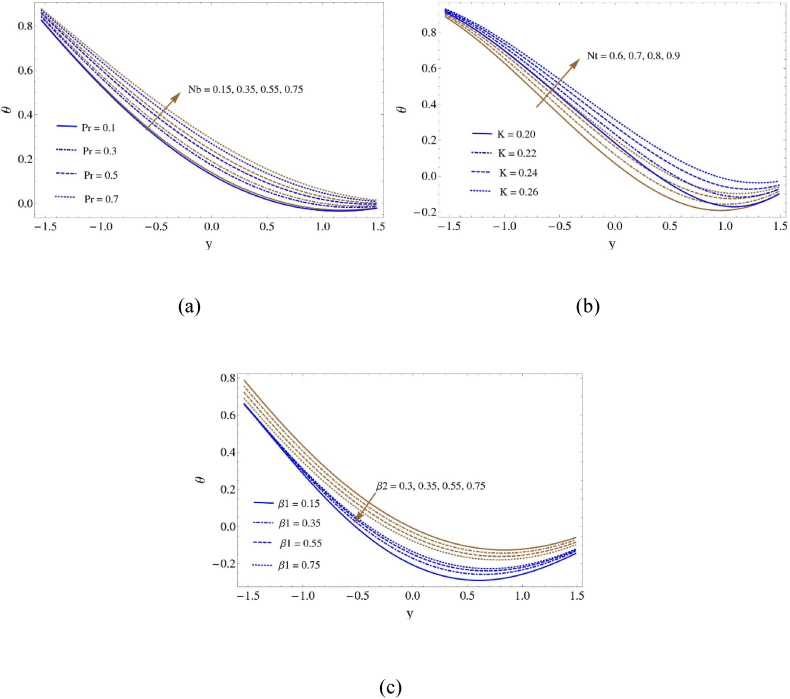
Heat profile (a) for Nb and Pr, (b) for K and Nt and (c) for $\beta_{1}$ and $\beta_{2}$

By looking at Fig. 3, we can observe how temperature profiles respond in Fig. 3(a and b) as higher values of the Thermophoresis parameter (Nt), Brownian number (Nb), Darcy number (K), and Prandtl number (Pr) are obtained. As the boundary slip characteristic $\left(\beta_{1}\right)$ increases, temperature curves increase as well, as shown in Fig. 3(c), which further indicates that increasing the thermal slip characteristic $\left(\beta_{2}\right)$ leads temperature curves to decrease. Greater value of Nt and Nb, respectively, indicate stronger thermophoretic and Brownian motion effects. Thermophoresis is the term used to describe how particles move in response to temperature gradients, whereas Brownian motion describes the random motion of particles resulting from collisions with fluid molecules. As Nt and Nb increase, particle motion becomes more important and enhances fluid mixing and dispersion. Temperature profiles can be impacted by the improved mixing because it disperses heat throughout the system more efficiently. While the Darcy number (K) indicates the ratio of viscous forces to inertial forces in porous media flow, the Prandtl number (Pr) describes the ratio of momentum diffusivity to thermal diffusivity in a fluid. Higher values of K and Pr may lead to different features of heat transport. Increased K denotes a predominance of viscous effects, which can affect fluid flow patterns and heat distribution in porous media. Increased Pr values imply that momentum diffuses more readily than heat, which could affect the thermal boundary layer and temperature gradients in the fluid. An increase in the boundary slip characteristic (β₁) can result in reduced resistance to fluid flow at the solid-fluid interface, thereby facilitating smoother flow and enhanced heat transfer. Temperature curves rise as a result, as shown in Fig. 3(c). Conversely, increasing the thermal slip characteristic (β₂) tends to rise in temperature transfer at the boundary and a decrease in the temperature gradient near the surface. This results in a decline in the temperature curves, as seen in the image.

**Fig. 4:**
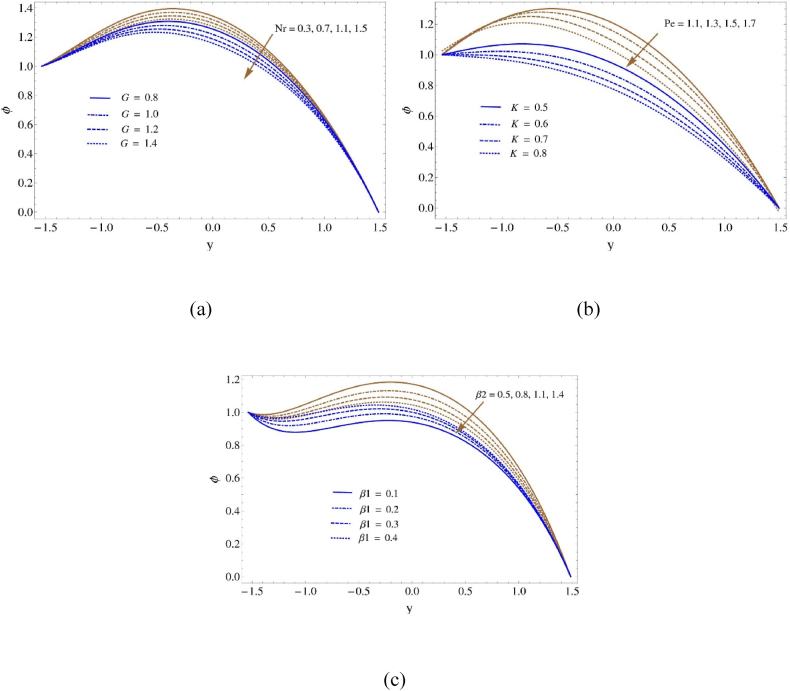
Volume friction of nanoparticale profile (a) for Nb and Pr, (b) for K and Nt and (c) for $\beta_{1}$ and $\beta_{2}$

We analyze nanoparticle concentration in detail in Fig. 4(a–c), providing explanations for its complex interactions with multiple physical factors. It becomes clear that a number of components have a significant connection to the behavior of nanoparticle concentration, providing insight into the intricate dynamics of the system. As the Boundary slip characteristic $\beta_{1}$ grows, so does the concentration of nanoparticles. On the other hand, the Peclet number (Pe), Darcy number (K), buoyancy ratio (Nr), Grashof number (G), and heating slip characteristic $\beta_{2}$ indicate an adverse correlation with nanoparticle concentration. This inverse relationship reveals how changes in these parameters which are related to heat transfer and convection characteristics affect the fluid's nanoparticle dispersion and deposition. The inverse correlation highlights how these essential mechanical properties could affect the concentration of nanoparticles. As the border slip characteristic $\beta_{1}$ increases, the concentration of nanoparticles also increases. The boundary slip characteristic is a measure of the fluid's slip at the solid-fluid interface, which indicates reduced friction between the fluid and the boundary. Because there is less friction between the nanoparticles and the fluid, they adhere to surfaces more easily and can be suspended in higher concentrations. Conversely, the concentration of nanoparticles is negatively correlated with the Grashof number (G), buoyancy ratio (Nr), Peclet number (Pe), Darcy number (K), and heating slip characteristic $\beta_{2}$.These factors are related to heat transmission and convection characteristics of the fluid. Higher Pe values suggest enhanced advective transport relative to diffusive transport, which could lead to better nanoparticle deposition and dispersion. However, this may also lead to increased mixing and dilution of the nanoparticle concentration. Similarly, higher K (Darcy number) values indicate stronger viscous effects, which may obstruct the fluid's ability to disperse nanoparticles. The buoyancy ratio (Nr) and Grashof number (G) show that buoyancy-driven flow is predominant.

**Fig. 5:**
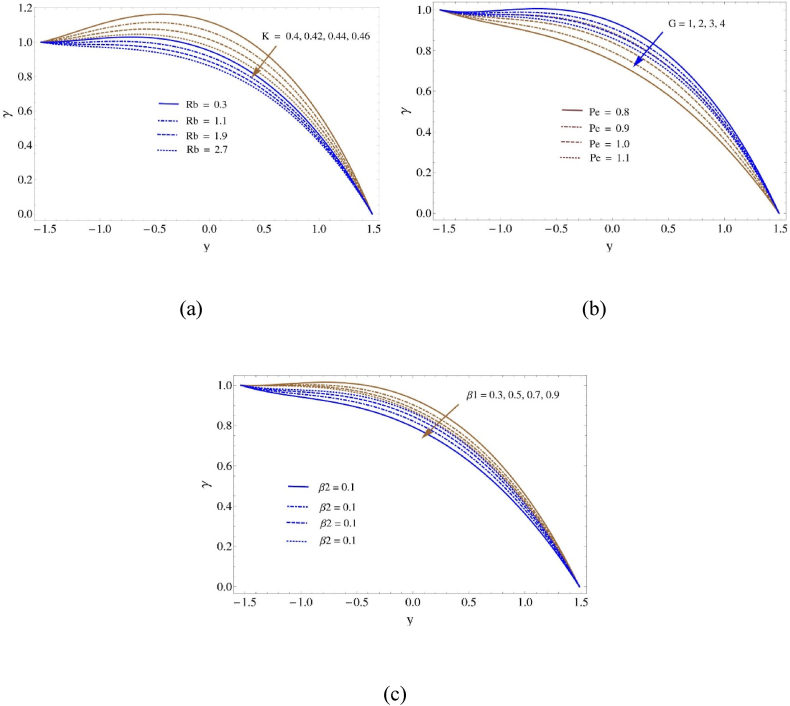
Density of motile microorganisams profile (a) for K and Rb, (b) for Pe and G and (c) for $\beta_{1}$ and $\beta_{2}$

The special behavior of wandering microorganisms is illustrated in Fig. 5. A comprehensive examination of microorganism concentrations in Fig. 5(a–c) detects significant deviations and illustrates the complex interplay between significant factors and microbial dynamics. Changes in the Peclet number (Pe) and Thermal slip characteristic $\left(\beta_{2}\right)\text{.}$ have been observed to increase the density of microorganisms. On the other hand, the association between the quantity of microorganisms and the bioconvection Rayleigh number (Rb), Darcy number (K), Grashof number (G), and boundary slip characteristic $\left(\beta_{1}\right)$ is opposite. The sensitivity of the bacterial concentration to variations of specific physical parameters is emphasized by this negative association. It has been observed that a higher microorganism density is correlated with increases in the Peclet number (Pe) and thermal slip characteristic (β₂). The Peclet number, which indicates if diffusive or convective processes predominate in fluid flow, characterizes the ratio of diffusive to advective transport. A higher Pe indicates greater advective movement, which could help with microorganism concentration and dispersion. Moreover, an increase in the thermal slip characteristic (β₂) may enhance the heat transfer at the boundary, creating an atmosphere that favors the growth and survival of microorganisms. However, there is an inverse relationship between these parameters (β₁,G,K, and Rb) and the density of microorganisms. The bioconvection Rayleigh number (Rb) is the ratio of buoyant forces to viscous forces in bioconvection flows, which is caused by microorganisms causing fluid motion. Higher Rb values suggest greater buoyancy-driven movement, which may inhibit microbial aggregation or facilitate dispersion. Likewise, buoyancy effects and fluid movement are influenced by the Grashof number (G) and Darcy number (K), which have the potential to disperse microbes throughout the system. Furthermore, increasing the boundary slip characteristic (β₁) at the solid-fluid interface may facilitate the dispersion of microorganisms rather than their aggregation.

**Fig. 6:**
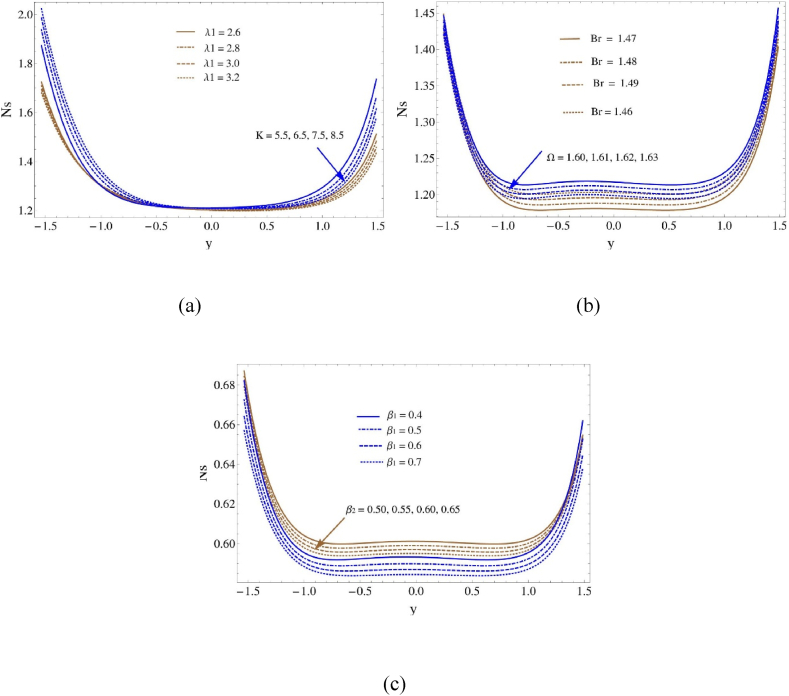
Entropy profile (a) for $\lambda_{1}$ and K, (b) for Ω and Br and (c) for $\beta_{1}$ and $\beta_{2}$

Fig. 6(a–c) indicate how distinct flow parameters affect the behavior of entropy formation or decrease. The association between entropy generation and the Brinkman number (Br) has been shown in Fig. 6(a), where it can be seen that it increases as the Brinkman number grows. This indicates that there are more dissipative effects in the flow as the Brinkman number rises. On the other hand, when the concentration difference parameter (Ω) increases compared to the concentration, a reduction is seen. Entropy generation grows with an increase in the Darcy number (K) before to y=−0.2 and drops later, as Fig. 6(b) displays. On the other hand, the height of the entropy generation curves decreases with an increase in the ratio of relaxation to retardation times $\left(\lambda_{1}\right)\text{.}$ The behavior of the entropy function of production with respect to both thermal slip characteristic $\left(\beta_{2}\right)$ and boundary slip characteristic $\left(\beta_{1}\right)$ is appeared in Fig. 6(c). It is obvious that as the boundary slip characteristic $\left(\beta_{1}\right)$ and thermal slip characteristic $\left(\beta_{2}\right)$ grow, entropy generation reduces. This finding suggests that increased slip at the boundary leads the flow to get less irreversible, most likely as a result of decreased fluid-wall contacts and the resulting energy dissipation. On the other hand, entropy generation decreases when the thermal slip characteristic $\left(\beta_{2}\right)$ grows. As a result, increased thermal slip at the boundary generally results in a reduced entropy generation, most likely as due to of improved thermal transfer mechanisms and lower thermal waste.

**Fig. 7:**
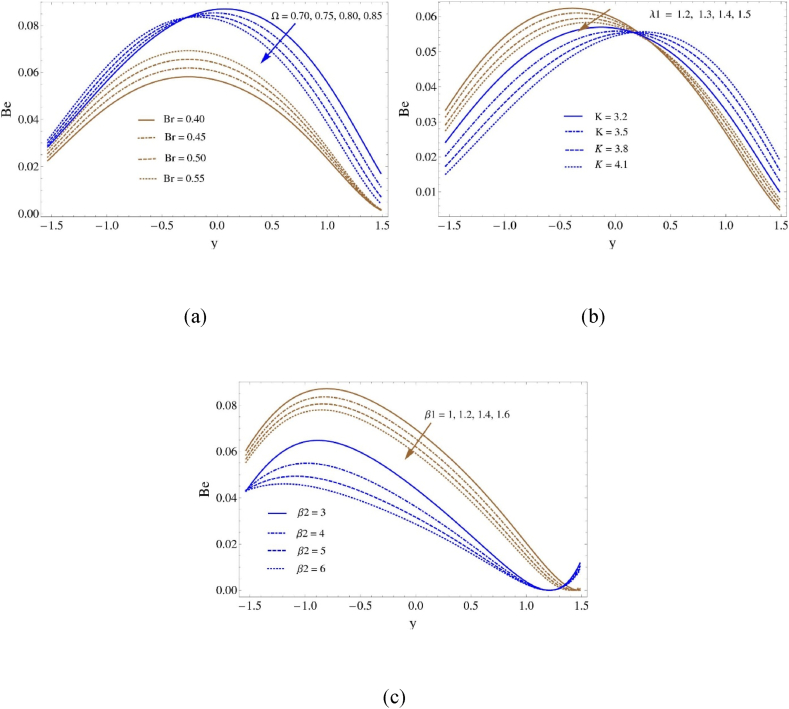
Bejan number profile (a) for Ω and Br, (b) for $\lambda_{1}$ and K and (c) for $\beta_{1}$ and $\beta_{2}$

Fig. 7 provides an analysis of the effects of Bejan number curves each of the following parameters: Brinkman number (Br), concentration difference parameter (Ω), Darcy number (K), boundary slip characteristic $\left(\beta_{1}\right)$ ratio of relaxation to retardation times $\left(\lambda_{1}\right)\text{,}$ and thermal slip characteristic $\left(\beta_{2}\right)$. As the concentration difference parameter (Ω) increases, the Bejan number profile grows for y<−0.4 and thereafter declines, shown in Fig. 7(a). In similar fashion, the Bejan number profile climbs in response to an increase in the Brinkman number (Br). As the proportion of relaxation to retardation times $\left(\lambda_{1}\right)$ and the Darcy number (K) grow in the region y<0.2, the Bejan number falls, whereas in the region y>0.2, it increases, displayed in Fig. 7(b). The influence of the thermal slip characteristic $\left(\beta_{2}\right)$ and boundary slip characteristic $(\beta_{1}$) on the Bejan number can be observed in Fig. 7(c). An increase in values of $\left(\beta_{1}\right)$ produces improved flow-wide momentum and energy transfer, which in turn decreases the creation of entropy. On the other hand, entropy generation decreases when the thermal slip characteristic $\left(\beta_{2}\right)$ increases.

**Fig. 8:**
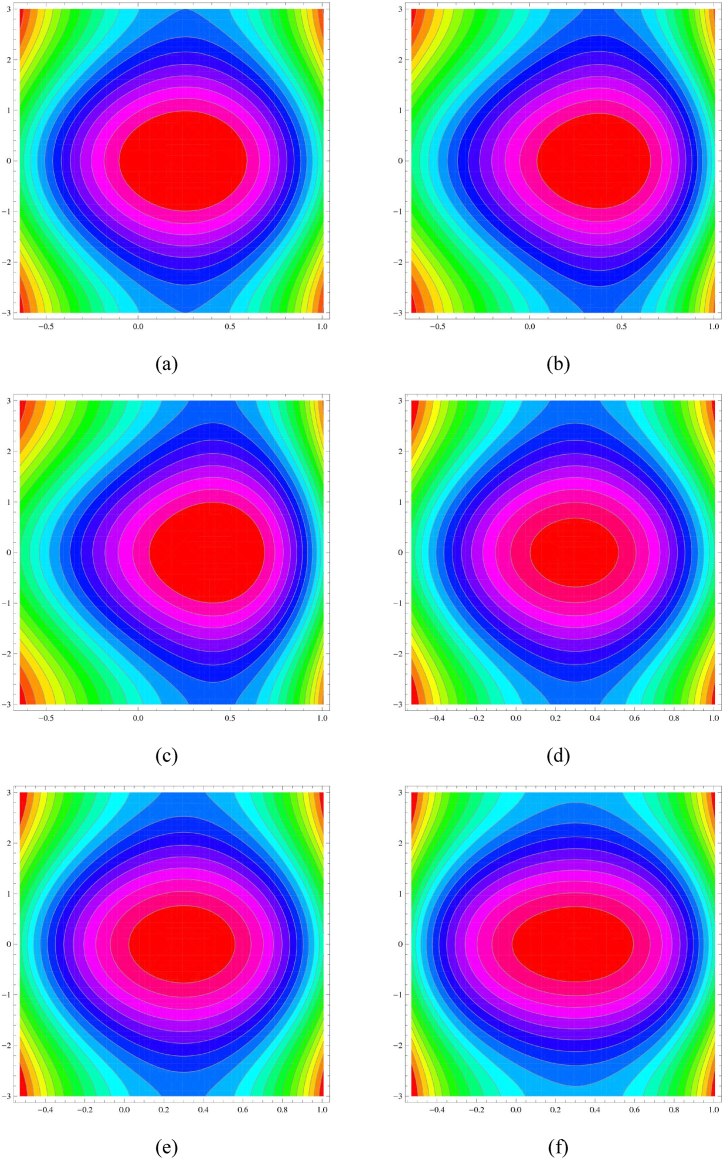
Stream line (a–c) for G and (d–e) for M

Fig. 8(a–f) shows an illustration of the system's velocity contours accounting for variations in the Grashof number (G) and the Hartmann number (M). This study illustrates the intricate relationships between these variables and how they effect the streamlines of the system. Fig. 8(a–c) illustrate how the velocity contours are impacted by (G). It is shown that the bolus moves to the right and that the size of the velocity contours decreases with an increase in (G). As (G) increases, it can be observed that the fluid moves more quickly to the right because the buoyancy force becomes more significant. Moreover, velocity contour sizes decrease as (G) increases because of a faster fluid mixing brought on by increased convective heat transfer. The impact of the Hartmann number (M) on velocity is depicted in Fig. 8(d–f). The bolus demonstrates how (M) affects velocity contours and how its size increases with (M). The figure illustrates how (M) affects velocity contours, with the size of the bolus growing as (M) increases. This phenomenon can be explained by the magnetic field produced by the Hartmann number, which acts as a Lorentz force on the conducting fluid and stifles fluid motion perpendicular to the magnetic field lines. Consequently, as (M) rises, the impact of the magnetic field increases, restricting fluid movement and resulting in larger velocity contours.

## 5 Conclusions

In their most recent work, authors have discovered the solutions for the Jeffrey nanofluid model's peristaltic flow in a channel, as well as the impacts of microbes and magnetic fields. Lubrication theory is applied in a moving laboratory frame to create a mathematical model. For the momentum and energy equations, boundary conditions are regarded as boundary and thermal slip, whereas no-slip bounds are taken for the concentration of nanoparticles and the microbe profile. Furthermore, the analysis covered the irreversibility impacts resulting from heat loss, viscosity, motion of nanoparticles, and movements of microorganisms within the flow. Stream and thermal lines have been included to provide a virtual three-dimensional view of the thermal transfer and flow pattern. The investigation described here produced several important findings:

• It is found that the production of entropy is inversely proportional to (Ω) and directly associated with the parameters $\left(B_{r}\right)$, (Γ). Furthermore, the production of entropy rises on the right side and decreases in the left when Hartman number (M) grows.
• It is evaluated that when slip factor grows then entropy analysis velocity profile, concentration of nanoparticles, and motile microorganism's density also enhance but the profile of Bejan number falls consequently.
• It is analyzed that the boundary slip parameter $\left(\beta_{1}\right)$ causes grow in heat.
• Graphical representations imply that the concentration of microorganisms and Bejan factor increases along with rise in $\left(\beta_{2}\right)$, moreover the nano-particles volume concentration and entropy generation profile decline.
• It is crucial to note that the asymmetry in the channel's configuration causes the rate of flow and heat exchange to fluctuate randomly in the left and right sections of the conduit.
• It is evaluated that the stream boluses are expanded by the increasing impact of Grashof number and the Hartman number.
• It is calculated that thermal lines contracted by the Brinkman parameter but expanded for the Jeffrey fluid influence.

## Future directions

This work can be extended in the future by include electroosmotic impacts and for other viscoelastic fluids. However an experimental research on the topic will add significant contribution in the field.

**Table:** Nomenclature

Symbols with SI units	Name of parameter
$W_{c}$ [m/s]	Maximum cell swimming speed
$\bar{T}$ [K]	Local temperature
$\rho_{f}$ [Kg/m^3^]	Fluid density
μ [Pa.s]	Dynamic viscosity
α [m^2^/s]	Thermal diffusivity
$B_{0}$ [Tesla]	Magnetic field
g [m/s^2^]	Acceleration
k [W/mK]	Thermal conductivity
b	Chemotaxis constant
$\bar{n}$	Concentration of motile microorganisms
$\rho_{p}$	Concentration nanoparticle
$D_{B}$	Brownian diffusion coefficient
$D_{T}$	Thermophoretic diffusion coefficient
$\bar{x},\bar{y}$	Coordinates
Dimensionless parameters	
φ	Volume fraction of nanoparticles
M	Hartmann factor
$N_{b}$	Brownian motion factor
$N_{r}$	Buoyancy ratio
$N_{t}$	Thermophoresis factor
$R_{b}$	Rayleigh number
$P_{r}$	Prandtl number
$\sigma_{1}$	Bioconvection constant
$P_{e}$	Peclet number
Ω	Density difference factor
Γ	Proportion of heat density factor
$\beta_{1}$	Slip boundary characteristic
$\beta_{2}$	Temperature slip characteristic
Λ	Temperature difference factor

## Data availability

No data was used for the research described in the article.

## CRediT authorship contribution statement

**Arshad Riaz:** Formal analysis. **Muhammad Dil Nawaz:** Writing – original draft, Methodology. **Muhammad Naeem Aslam:** Investigation. **Sami Ullah Khan:** Formal analysis. **Shafiq ur Rehman:** Software, Resources. **Ghaliah Alhamzi:** Writing – review & editing.

## Declaration of competing interest

The authors declare that they have no known competing financial interests or personal relationships that could have appeared to influence the work reported in this paper.
